# Negative correlation between leftward bias in line bisection and schizotypal features in healthy subjects

**DOI:** 10.3389/fpsyg.2013.00846

**Published:** 2013-11-14

**Authors:** Michele Ribolsi, Giulia Lisi, Giorgio Di Lorenzo, Giuseppe Rociola, Cinzia Niolu, Alberto Siracusano

**Affiliations:** Clinica Psichiatrica, Dipartimento di Medicina dei Sistemi, Università degli Studi di Roma Tor VergataRome, Italy

**Keywords:** pseudoneglect, line bisection, mental number line, schizotypy, psychosis

## Abstract

**Introduction:** Recent studies have found a lack of normal pseudoneglect in schizophrenia patients and in their first degree relatives. Similarly, several contributions have reported that measures of schizotypy in the healthy population may be related to signs of right-sided lateralization, but most of these studies differ greatly in methodology (sample size, choice of schizotypy scales, and laterality tasks) and, consequently, the results cannot be compared and so definitive conclusion cannot be drawn. In this study, our purpose is to investigate whether some tasks of spatial attention may be related to different dimensions of schizotypy not only in a larger sample of healthy subjects (HS), but testing the same people with several supposedly related measures several times.

**Materials and Methods:** In the first part of the study (Part I), the performance on “paper and pencil” line bisection (LB) tasks in 205 HS was investigated. Each task was repeated three times. In the second part of the study (Part II), a subgroup of 80 subjects performed a computerized version of the LB test and of the mental number line bisection (MNL) test. In both parts of the study, every subject completed the 74-item version of the Schizotypal Personality Questionnaire (SPQ) and the Edinburgh Handedness Inventory (EHI).

**Results:** In both parts of the study, high scores on the subscale “magical thinking” of SPQ have resulted in being closely linked to a decreased pseudoneglect as assessed by the LB task. On the contrary, right handedness is related to an increased leftward bias at the same task. No association was found between MNL and the other variables.

**Discussion:** The main finding of this study is that a decreased spatial leftward bias at the LB task correlates with positive schizotypy in the healthy population. This finding supports the hypothesis that a deviation from leftward hemispatial visual preference may be related to the degree of psychosis-like schizotypal signs in non-clinical population and should be investigated as a possible marker of psychosis.

## Introduction

Lateralization of cognitive functions between the right and left hemispheres is known to be a prominent feature of the human brain (Kosslyn, 1987; Hellige, 1990; Gazzaniga, 2000; Crow, 2010). For instance, it is known that the cortical networks of the right hemisphere involving the posterior parietal cortex play a dominant role in visuo-spatial attention, so that right hemisphere lesions often induce visuo-spatial neglect, a severe neurological disorder characterized by failure to acknowledge or explore stimuli presented to the contralesional side of space (Bisiach and Luzzatti, 1978; Heilman et al., 2000; Vallar et al., 2003). The most commonly used technique for detecting the presence of unilateral spatial neglect is the line bisection (LB) test: the patient is asked to place a pencil mark at the center of a series of horizontal lines. Displacement of the bisection mark toward the side of the brain lesion is interpreted as a symptom of neglect (referred to as perceptual neglect). Moreover, the phenomenon known as “pseudoneglect” (Bowers and Heilman, 1980) refers to the systematic leftward misbisection of horizontal lines made by neurologically intact observers. The magnitude of this bisection error is much smaller than in neglect patients but studies have widely shown that neglect and pseudoneglect are closely related (McCourt and Jewell, 1999) and possess similar susceptibilities to a variety of modulating factors. For example, both the magnitude and the direction of the bisection errors in pseudoneglect are modulated by stimulus or task factors (e.g., line length, line location, task instructions) (McCourt and Jewell, 1999; Fink et al., 2002) which may also influence the magnitude and the direction of the visual neglect (Marshall and Halligan, 1989; Seki et al., 1999). There is also evidence supporting the existence of both representational neglect and pseudoneglect, which can be detected through the mental number line bisection (MNL) test, in which numbers are conceived as falling along a mental number line spatially oriented from left to right and the subject is asked to bisect a numerical interval (Heilman et al., 2000; McGeorge et al., 2007). The LB test and the more recent MNL bisection test (Bisiach and Luzzatti, 1978) have different underlying mechanisms, the MNL is in fact mostly related to a purely abstract non-spatial representation of an imaginary number line (Aiello et al., 2013). Different anatomical areas are thought to be involved in these tasks: neuroimaging studies demonstrated that while physical LB depends on the striate, the extrastriate visual cortex and the inferior and superior parietal lobe, comparative judgments of numeric quantities activate prefrontal areas (Doricchi et al., 2005; Tian et al., 2011).

Interestingly, various psychopathological conditions may influence the expression of pseudoneglect (Rao et al., 2010). To our knowledge, in the context of schizophrenia research, six studies (Mather et al., 1990; Barnett, 2006; Michel et al., 2007; Zivotofsky et al., 2007; McCourt et al., 2008; Ribolsi et al., 2013) have investigated perceptual pseudoneglect and only three studies representational pseudoneglect (Cavezian et al., 2007; Tian et al., 2011; Ribolsi et al., 2013).

Concerning representational pseudoneglect, Cavezian and colleagues found an exaggerated leftward bias in the MNL of the schizophrenia patients (SCZ) in comparison to the healthy subjects (HS) (Cavezian et al., 2007), while two more recent studies found no difference between the two groups (Tian et al., 2011; Ribolsi et al., 2013).

However, concerning the relationship that occurs between schizophrenia and perceptual pseudoneglect, two studies reported a leftward bias in the SCZ sample (Mather et al., 1990; Michel et al., 2007) while all the others have provided evidence of a significant lack of leftward bisection error in the LB test (Barnett, 2006; Zivotofsky et al., 2007; McCourt et al., 2008; Ribolsi et al., 2013). This result has been hypothesized as being linked to a reduced or reversed brain asymmetry with a deficit of right hemisphere functions (Michel et al., 2007; Zivotofsky et al., 2007; McCourt et al., 2008; Rao et al., 2010; Ribolsi et al., 2013) and in particular of the right parietal cortex in SCZ (Petty, 1999; Malhotra et al., 2009; Venkatasubramanian et al., 2011). Intriguingly a dysfunction of this area has been linked to schizophrenia in a structural, neurophysiological and functional way (Zhou et al., 2007; Kato et al., 2011; Venkatasubramanian et al., 2011). Interestingly, in a recent study conducted by our research group, selective transcranial direct current stimulation (tDCS) of right posterior parietal cortex was able to determine a partial correction of the lack of leftward bias in a group of medicated SCZ, confirming the hypothesis of the involvement of this area in the onset of this phenomenon (Ribolsi et al., 2013).

In this study, our purpose is to investigate whether the LB and MNL performances may be related to different dimensions of schizotypy in a large sample of HS. In particular, our purpose is to investigate the hypothesis of a continuum between schizophrenia and schizotypy not only on a phenomenological and genetic level (Nelson et al., 2013) but also in measures of visuo-spatial attention and lateralization.

Schizotypy is a psychological construct that describes temporally stable personality characteristics and specific perceptions, cognition, beliefs, and experiences that are phenomenologically similar to, but less severe than, the symptoms of schizotypal personality disorder and schizophrenia (Meehl, 1962; Shaw et al., 2001). This condition resembles schizophrenia not only in terms of observable symptoms but also in the underlying multidimensional structure (Gruzelier, 1996). There is a wide variety of questionnaires designed for the assessment of schizotypy: the most widely used are the Wisconsin-Madison scales (Chapman and Chapman, 1980), the Schizotypal Personality Questionnaire (SPQ) (Raine, 1991) and the Oxford-Liverpool Feelings and Experiences Questionnaire (O-LIFE) (Mason and Claridge, 2006).

Unfortunately, till now the studies on the link between schizotypy and functional hemispheric asymmetry have been inconclusive, with some of them reporting a right-over-left hemisphere shift (Kravetz et al., 1998; Suzuki and Usher, 2009), others a left-over-right hemisphere shift (Mason and Claridge, 1999; Liouta et al., 2008), and yet others finding no relation between schizotypy and laterality (Najt et al., 2012). All these studies used different measures of laterality (language and visuo-spatial attention tasks).

In the debate concerning the relation between schizotypy and measures of laterality (Schofield and Mohr, 2013) only a few studies have examined whether pseudoneglect is related or not to a specific dimension of schizotypy. Results are controversial. Using a tactile rod bisection task, Brugger and Graves (1997) highlighted a right-sided inattention and reported a significant association between the size of this right-sided inattention and high magical ideation scores in male participants. Nalcaci et al. (2000), evaluating performances on Corsi's Block-Tapping Test, also reported an association between right hemispatial inattention and high magical ideation scores. These findings have been interpreted as supporting the notion that a deficit in left hemispheric functioning underlies schizotypy. Contradicting results are reported by Liouta and colleagues, who reported an association between schizotypy and rightward hemispatial bias using two different spatial behavior tasks (Liouta et al., 2008). Interestingly, Mohr and colleagues, using three different kinds of spatial behavior tasks (LB “paper and pencil,” whole-body turns, and veering behavior when attempting to walk in a straight line while blindfolded), reported that high scores in magical ideation scales were linked, on the one hand, to right-sided inattention at lateralized whole-body movement tasks (turning and veering) and on the other hand to a lack of pseudoneglect in conventional “paper and pencil” LB tasks (Mohr et al., 2003).

In conclusion, as shown in Table 1, what emerges from today's literature is a multifaceted research panorama in which the studies conducted are very different in methods (choice of schizotypy scales and laterality tasks) and, consequently, the results and conclusions are not fully comparable. In this context, the aim of our study is to investigate the supposed correlation between different dimensions of schizotypy, LB and MNL performances not only in a larger sample of HS, but testing the same people with several supposedly related measures several times. Given the current literature, we would expect to find a relation between a decreased pseudoneglect and the degree of schizotypal traits in HS. Furthermore, in our study we hypothesize that, unlike the LB test, the performance in the MNL test is not influenced by schizotypy, probably because of its non-spatial origin (Van Dijck et al., 2011).

**Table 1:** **Summary of the studies that investigated schizotypy and spatial tasks**.

**Study**	**Subjects**	**Spatial tasks**	**Measures of Schizotypy**
Brugger and Graves, 1997	40 HS	Rod-centering task	Magical ideation scale
	(20 M; 20 F)		
Nalcaci et al., 2000	98 HS	Modified Corsi's block-tapping test	Magical ideation scale
	(66 M; 32 F)		
Kalaycioglu et al., 2000	76 HS	Modified Corsi's block-tapping test	Magical ideation scale
	(38 M; 38 F)		
Taylor et al., 2002	40 HS	Implicit line bisection (Rey-Osterrieth complex figure's copy task)	Magical ideation scale
	(40 M)		
Mohr et al., 2003	36 HS	Line Bisection “paper and pencil”	Magical ideation scale
	(16 M; 20 F)	Turning behavior	
		Veering behavior	
Gooding and Braun, 2004	50 Schizotypic Students	Rey-osterrieth complex figure test	Chapman scales
	(50 M)		
Liouta et al., 2008	40 HS	Line Bisection “paper and pencil”	O-LIFE (Oxford-Liverpool inventory of feeling and experiences)
	(40 M)	Whole-body movement task	
Brugger et al., 2010	40 HS	Number line bisection	Magical ideation scale
	(20 M; 20 F)		

## Materials and methods

### Subjects

In Part I, 205 HS were recruited [136 women; age (*SD*): 34.78 (12.74)], while in Part II 80 HS were recruited [46 women; age (*SD*): 32.23 (11.05)].

The subjects were recruited by personal contact and flyers posted at the University Hospital of Tor Vergata, Rome. All the subjects had a university education.

In both parts, each subject underwent a specialistic neurological and psychiatric consultation before recruitment. Subjects were excluded from participating if they exhibited any neurological or ophthalmological disorders (as assessed by a careful neurological examination), a history of head trauma (as reported by the subjects), or if they met criteria for substance dependence within the previous 6 months, or substance abuse within the month preceding testing. Moreover, a diagnosis of any psychiatric disorder was excluded by means of consultations with physicians and the Structured Clinical Interview for *Diagnostic and Statistical Manual of Mental Disorder, Fourth Edition*. All subjects gave written informed consent for the study. The experimental procedures used were approved by the local Ethics Committee and were carried out in accordance with the Declaration of Helsinki. Finally, for each subject handedness was ascertained by the Edinburgh Handedness Inventory (EHI) (Oldfield, 1971). The EHI is a valid and reliable quantitative measurement tool that assesses a participant's hand, eye and foot preference for 12 tasks (Ransil and Schachter, 1994). Finally, each subject underwent the 74-item version of the SPQ to evaluate the presence of schizotypal traits. The SPQ is a 74-item, forced choice, self-report questionnaire, which yields nine subscales designed to give a dimensional assessment of the Schizotypal Personality Disorder features listed in the DSM-III-R (APA, 1987): ideas of reference, excessive social anxiety, odd beliefs, or magical thinking, unusual perceptual experiences, odd, or eccentric behavior, no close friends, odd speech, constricted affect, suspiciousness (Raine, 1991). In our study, we used the Italian version validated by Fossati et al. (2003).

Although several factor analytic studies of this measure have been conducted (Linscott, 2013), we chose to use the SPQ subscales in order to investigate every specific feature of schizotypy according to the DSM-III-R rather than grouping the different dimensions. Similarly, various previous studies have selectively investigated the relation between a single specific dimension of schizotypy and measures of pseudoneglect (Brugger and Graves, 1997; Kalaycioglu et al., 2000; Taylor et al., 2002; Mohr et al., 2003; Brugger et al., 2010). In this regard, recent studies have reported that the single SPQ subscales may be useful in screening for schizotypal traits in the general population (Bedwell et al., 2013; Salokangas et al., 2013).

### Part I

Subjects were seated comfortably at a writing table and the pages (size A4) containing the tasks were placed in front of them. They were told to follow the instructions written in Italian at the top and then to fixate the line in the paper. All tasks were to be performed using the dominant hand. The lines to be divided in the bisection task were centered on each page and located below the midline. The lines were 125 mm-long black lines on an otherwise blank white page. Every page instructed the subject to “divide the line in half as accurately as possible.” Participants were asked to bisect each line into two equal lengths using a pencil. In order to compute scores, each line was measured to the nearest millimeter. The deviation from the center of the line was calculated as the absolute error in mm. Negative values indicate leftward bias and positive values a rightward bias. The task was repeated three times on three different days (a total of nine lines for each subject) and the mean deviation was calculated.

### Part II

In this part of the study, the subjects enrolled underwent a computerized version of the LB test and of the MNL test. E-Prime® (version 2.0, Psychological Software Tools, Inc.; http://www.pstnet.com) software was used to create computerized versions of the two pseudoneglect protocols. Stimulus presentations and data collection were performed on a 15.4″ laptop computer screen.

#### Line bisection

Visual stimuli consisted of black 1 mm-thick horizontal lines transected by a 1 mm-thick and 1 cm-long vertical bar, presented on a white background with the transector positioned exactly in the center of the screen. A modified version of LB created by Fierro et al. (2000) was performed to detect LB in medicated psychiatric patients. Stimuli were presented for 750 ms. Three lines of 15 cm were presented, differing in the position of the transector (at midpoint, rightward, or leftward). Subjects were given 30 trials in random order, 10 with the transector at the exact center (7.5 cm), 10 with a rightward transector at 8.0 cm and 10 with a leftward transector at 7.00 cm. Interstimulus intervals were 3750 ms.

Subjects were seated at a distance of 45 cm from the laptop screen and were asked to focus on a centrally positioned fixation cross that disappeared as soon as the three numbers were presented.

Participants judged the position of the transector in pre-bisected lines by pressing one of three buttons with the right index, middle, or ring finger for “left,” “equal,” or “right” responses. The performance of the subject on each trial was scored as follows: 0, correct response; 1, if the subject judged the transector to be right of its real position; –1, if the subject judged the transector to be left of its real position.

#### Mental number line

Stimuli (integers from 1 to 99) consisted of 30 different one- and two-digit number triplets, constituted by a middle number and two outer numbers defining a number interval for each side. The three number stimuli were spaced 25 mm apart. The numerical distance between the middle number and the outer numbers was equal (bisectable triplets: e.g., 2_9_16), bigger on the right side (e.g., 9_15_6), or on the left side (e.g., 9_19_11) in an equal number of trials. Triplets that are part of a multiplication table were not included. Stimuli were presented for 750 ms, with the middle number exactly in the center of the 15.4″ laptop screen. The intertrial interval was 3750 ms. Subjects were seated at a distance of 45 cm from the screen and were asked to focus on a central fixation cross that disappeared as soon as the three numbers were presented.

Participants judged the magnitude of the middle number in relationship to the outer ones by pressing one of three buttons with the right index, middle, or ring finger for “left,” “equal,” or “right” responses. The performance of the subject on each trial was scored as follows: 0, for a correct response; 1, if subjects judged the middle number nearer to the right number of the triplet;

−1, if subjects judged the middle number nearer to the left number of the triplet.

### Statistics

The normal distribution of the pseudoneglect indices was evaluated with Shapiro-Wilk *W*-test. In the case of a non-normal distribution, a logarithmic transformation was performed prior to statistical analysis to achieve an appropriate equivalence to a normal distribution (Shapiro-Wilk test, *p* > 0.05 consistently).

Stepwise multiple linear regression analysis was used in order to investigate the predictors of LB index (Part I and II) and MNL index (Part II). We built regression models with LB (or MNL) index as the dependent variables (DVs); as possible independent predictors we entered in the regression models only the variables that were significant in the univariate analyses (*t*-test for independent sample and Pearson's product-moment correlation). As the presence of outliers can increase type I error rate in regression analysis, Mahalonobis' Distance (MD) was used to identify potential multivariate outliers. As effect size measures, the Beta standardized coefficient (β) and *R*^2^ change were also reported to evaluate the degree of association between the significant independent predictor(s) and the DV. Statistical significance was set at *p* < 0.05.

## Results

All pseudoneglect indices in the two parts of the study had a normal distribution (Experiment 1: LB index, *W* = 0.994, *p* = 0.813; Experiment 2: LB index, *W* = 0.989, *p* = 0.282; MNL index, *W* = 0.985, *p* = 0.120).

**Figure 1:**
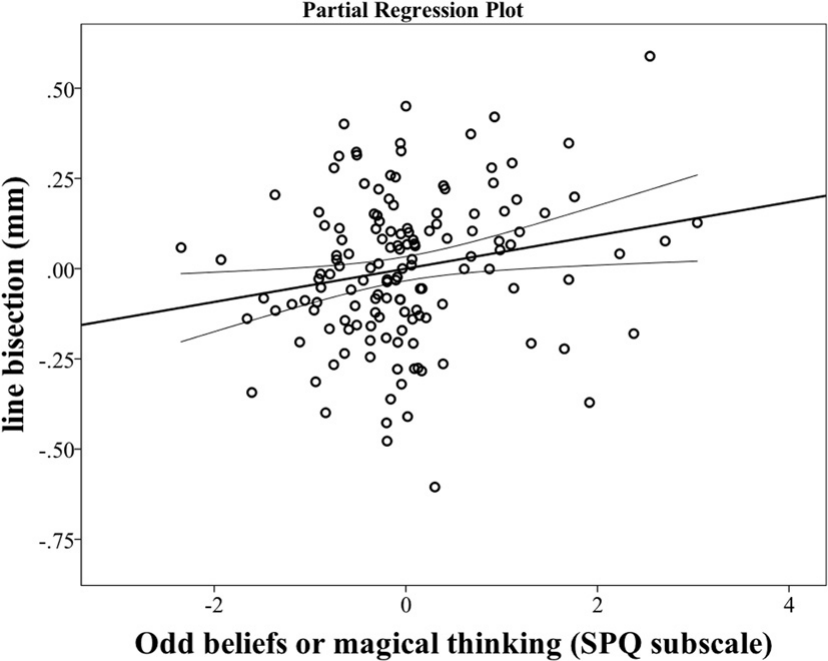
**The line bisection (LB) index was independently predicted by “Odd beliefs or magical thinking” SPQ scale (*p* < 0.0001, β = 0.273).** In the figure, the partial regression plot^a,b^ outlines the single relation between the “Odd Beliefs or Magical Thinking” subscale score (independent predictor) and LB index (dependent variable). ^a^This plot shows the real and specific effects of one independent predictor (“Odd Beliefs or Magical Thinking”) on dependent variable (LB), controlling (or regressed) independently for the other predictor. More in detail, the scatterplot (based on least squares fitting) displays the residuals (or errors: correspond to unexplained or residual variation between the observed values of the variable and the values suggested by the regression model) of each independent predictor variable and the residuals of the dependent variable, after the effect of other predictor has been removed separately on the two variables. ^b^Bold line is the fit line, representing the trend of the data. This linear regression (based on least squares method) line fits, in the best way, all the data points in the graphs. Thin lines show the CI_95_ for mean.

For the analysis of the Part I, we entered gender, age, EHI and “Odd beliefs or magical thinking” SPQ scale in the regression model as possible predictors of LB index (see Tables 1, 2 in Supplementary Materials). MD critical value of chi-square distribution, for degrees of freedom = 3 and *p* < 0.001, was 16.27. The regression model was significant [*F*_(3, 201)_ = 12.791, *p* < 0.0001] and explained 12% (adjusted *R*^2^ = 0.119) of LB index variance. Observed statistical power of the regression model was 0.997. No multivariate outliers were detected in the model (highest MD value: 8.739). The LB index was independently predicted by “Odd beliefs or magical thinking” SPQ scale (*p* < 0.0001, β = 0.273, *R*^2^ change = 0.076) (Figure 1) and EHI (*p* < 0.0001, β = −0.231, *R*^2^ change = 0.052) (Table 2A). A decreased pseudoneglect was related to an increase of odd beliefs or magical thinking scores and a decrease of handedness score (indicating a leftward/mixed hand attitude).

Table 2**Results of regression models in Experiment 1 (A) and Experiment 2 (B)**.

**Table:** **(A) Experiment 1. Dependent variable: LB index**.

**Independent variable**	**Beta**	***t***	***P***	**Model step**	***R*^2^ change**	***F* change *p*-level**	***F* to remove^a^/to enter^b^**	***p* to remove^c^/to enter^d^**
Odd beliefs or magical thinking	0.273	3.708	<0.0001	1	0.076	<0.0001	13.751^a^	<0.0001^c^
EHI	−0.231	−2.714	<0.0001	2	0.052	<0.0001	7.367^a^	<0.0001^c^
Age	0.111	1.421	0.158	−	−	−	2.216^b^	0.139^d^
Gender	−0.083	−1.048	0.297	−	−	−	1.288^b^	0.258^d^

*Regression model: adjusted R^2^ = 0.119; F_(3, 201)_ = 12.791; p < 0.0001*.

**Table:** **(B) Experiment 2. Dependent variable: LB index**.

**Independent variable**	**Beta**	***t***	***P***	**Model step**	***R*^2^ change**	***F* change *p*-level**	***F* to remove^a^/to enter^b^**	***p* to remove^c^/to enter^d^**
Odd beliefs or magical thinking	0.281	3.752	<0.0001	1	0.097	<0.0001	14.581^a^	<0.0001^c^
EHI	−0.242	−2.756	<0.0001	2	0.066	<0.0001	8.147^a^	<0.0001^c^

*Regression model: adjusted R^2^ = 0.141; F_(2, 77)_ = 11.801; p < 0.0001*.

For the analysis of the Part II, we build two regression models, respectively, in the first one LB index was the DV and MNL index in the second one.

We entered EHI and “Odd beliefs or magical thinking” SPQ scale in the first regression model as possible predictors of LB index (see Tables 1, 2 in Supplementary Materials). MD critical value of chi-square distribution, for degrees of freedom = 2 and *p* < 0.001, was 13.82. The first regression model was significant [*F*_(2, 77)_ = 11.801, *p* < 0.0001] and explained 14% (adjusted *R*^2^ = 0.141) of LB variance. No multivariate outliers were detected in the model (highest MD value: 9.572). The LB values was independently predicted by “Odd beliefs or magical thinking” SPQ scale (*p* < 0.0001, β = 0.281, *R*^2^ change = 0.097) and EHI (*p* < 0.0001, β = −0.242, *R*^2^ change = 0.066) (Table 2B).

As no association was found between MNL index and the other variables at the univariate level, no variable entered in the second regression model (MNL index as DV) (see Tables 1, 2 in Supplementary Materials).

## Discussion

In this study, we investigated whether some tasks of spatial attention may be related to the degree of schizotypal traits in the healthy population. In particular, our purpose was to investigate the relation between the single dimensions of the SPQ and some measures of both perceptual pseudoneglect (the LB task) and representational pseudoneglect (the MNL task).

### Part I

The main finding of Part I is that a deviation from the leftward bias on the LB task correlates with schizotypy in the healthy population, particularly with the dimension of “magical thinking.” To date, the current study is the largest one ever published on HS (as the observed statistical power of the regression model was 0.997).

Similarly, Liouta and colleagues found in a sample of forty right-handed HS a rightward bisection as a function of positive schizotypy (Liouta et al., 2008). These results contradict several previous findings which have shown a correlation between magical ideation and a leftward shift in spatial attention (Brugger and Graves, 1997; Kalaycioglu et al., 2000; Nalcaci et al., 2000; Taylor et al., 2002; Mohr et al., 2003; Brugger et al., 2010).

It is possible to hypothesize that different schizotypy questionnaires are potential contributors to the contradictory findings in the literature. This hypothesis is certainly undesirable and raises questions about the validity of the basic psychometric tool for assessing schizotypy: self-report questionnaires (Liouta et al., 2008). The majority of the previous studies on this topic used the Magical Ideation Scale (Brugger and Graves, 1997; Kalaycioglu et al., 2000; Taylor et al., 2002; Mohr et al., 2003; Brugger et al., 2010) while in the study by Liouta et al. (2008) schizotypy was assessed through the Oxford-Liverpool Inventory of Feelings and Experience (O-LIFE) (Mason et al., 1997; Mason and Claridge, 2006). This questionnaire produces scores for three main factors of schizotypy: positive, negative and cognitive disorganization. To assess schizotypy in our study, we used the Schizotypal Questionnaire (SPQ). Differently from the Magical Ideation and Perceptual Aberration scales, for example, which represent only single features of schizotypal personality, the SPQ contains all the subscales for all nine schizotypal traits according to DSM-III-R. Because of its high internal reliability and test-retest reliability the SPQ may be useful in screening for schizotypal personality disorder in the general population and also in researching the correlates of individual schizotypal traits (Raine, 1991).

In the present study, only the Magical Thinking subscale is related to LB performance. Very similarly, in the previous studies only the positive psychopathological domains of schizotypy correlated with the degree of pseudoneglect (Brugger and Graves, 1997; Kalaycioglu et al., 2000; Nalcaci et al., 2000; Taylor et al., 2002; Mohr et al., 2003; Liouta et al., 2008; Brugger et al., 2010). This is partially because the majority of these studies used the magical ideation scale in isolation (Brugger and Graves, 1997; Kalaycioglu et al., 2000; Taylor et al., 2002; Mohr et al., 2003; Brugger et al., 2010) and in another study none of the Chapman scales (magical ideation, perceptual aberration, social anhedonia, physical anhedonia) were related to hemispatial attention (Gooding and Braun, 2004). Conversely, Liouta et al. (2008) found that only positive schizotypy, and not cognitive disorganization and negative schizotypy, correlated with LB performance. Positive schizotypy (Ruhrmann et al., 2010; Barrantes-Vidal et al., 2013) and mild subthreshold psychotic symptoms (Yung et al., 2004) have a strong predictive value of proneness to psychosis and this may provide some explanation for the correlation between positive schizotypy (and the magical thinking dimension) and the well-observed rightward bias at the LB performance in schizophrenia patients (Barnett, 2006; Zivotofsky et al., 2007; McCourt et al., 2008; Rao et al., 2010).

Another important aspect to be considered is that spatial pseudoneglect is not a discrete measure, as it depends on several variables, such as the segment length and spatial dislocation of the stimulus (Balconi et al., 2012), the hand (Leonards et al., 2013), and the direction of performance of the endpoint task, i.e., left-to-right or right-to-left (Urbanski and Bartolomeo, 2008). Following this line of research, some studies have investigated the influence of different psychiatric disorders in LB performance. In particular, these previous studies have shown that whereas patients with affective disorders seem to have a leftward bias (He et al., 2010; Rao et al., 2010), patients with psychotic disorders show the opposite (Barnett, 2006; Zivotofsky et al., 2007; McCourt et al., 2008; Rao et al., 2010; Ozel-Kizil et al., 2012; Ribolsi et al., 2013). In this study, we suggest the hypothesis that, among the other variables, schizotypy, as a measure of proneness to psychosis, may influence the degree of pseudoneglect.

In this regard, the result of this part of the study is in line with our previous study which has shown that SCZ have an abnormal rightward bias in comparison with HS in the LB test (Ribolsi et al., 2013). Given the data from both of our studies, it is possible to hypothesize that besides a continuum of psychosis-like and schizotypal traits across both clinical and non-clinical populations, there is a corresponding continuum of deviation from the leftward bias in subjects with schizotypal traits to an abnormal clear rightward bias on the LB task in SCZ. Therefore, it is interesting to remember that schizotypy is presumed to reflect a genetically-determined disposition to schizophrenia (Meehl, 1962; Cadenhead and Braff, 2002). On the basis of a dimensional model of psychosis that assumes that pathological symptoms of schizophrenia lie on a continuum with psychosis-like schizotypal signs in non-clinical populations (Meehl, 1962; Eysenck and Barrett, 1993; Claridge et al., 1998; Van Os et al., 2000; Verdoux and Van Os, 2002), there is thought to be a link between schizotypy and schizophrenia not only on a phenomenological and genetic level but also in measures of visuo-spatial attention and lateralization.

Furthermore, in our previous study, we found that right parietal tDCS altered the performance of SCZ in the LB test with a partial correction of the rightward bias. We hypothesized that this correction could be due to an increase in the neural activity of the right PPC induced by tDCS (Ribolsi et al., 2013). In this regard, several studies reported the involvement of the right PPC in the phenomenon of visual neglect (Heilman et al., 2000; Vallar et al., 2003; Koch et al., 2008) and other authors have hypothesized that a right parietal dysfunction is responsible for the rightward bias in the LB test in schizophrenia (McCourt et al., 2008).

Similarly, abnormalities of hemispheric asymmetry assessed by right hemisphere tasks have been related to right hemisphere dysfunction in positive schizotypy (Jutai, 1989; Overby et al., 1989; Claridge and Beech, 1996; Mason et al., 1997; Nunn and Peters, 2001). On the basis of the hypothesis of the continuum between schizotypal traits and schizophrenia, we can hypothesize the involvement of the right parietal cortex dysfunction to explain the relationship between performance on the LB task and measures of schizotypy in the healthy population, but further studies are needed.

However, some recent studies have supported the hypothesis that schizotypy, and in particular magical ideation, may be related to reduced cerebral asymmetry for language (Crow, 1997; Barnett and Corballis, 2002), and that magical ideation and creativity are related to enhanced right hemisphere processing (Taylor et al., 2002; Weinstein and Graves, 2002). Recently, however, it has been shown that both magical ideation and creativity are negatively correlated with absolute hand preference but not with hand performance or with other signs of cerebral asymmetries, strongly contradicting the hypothesis of a neuropsychological explanation based on reduced single hemisphere dominance (Badzakova-Trajkov et al., 2011). Finally, other recent studies have focused their attention on brain measures other than those based on hemisphericity. In particular, subjects with high positive schizotypy show morphologic abnormalities in brain areas which have been studied also in high-risk mental state subjects and in schizophrenia, confirming that psychotic or psychotic-like experiences may have common neuroanatomical correlates across schizophrenia spectrum disorders (Modinos et al., 2010).

### Part II

In this part of the study, the subjects underwent a computerized version of the LB test and the MNL test.

MNL is a common technique for assessing the so-called “representational pseudoneglect.” In Western cultures, the mental representation of numbers takes the form of a number line along which magnitude is positioned in ascending order from left to right. Patients with right brain damage usually neglect smaller numbers while mentally setting the midpoint of number intervals (Vuilleumier et al., 2004; Umilta et al., 2009).

In our study, as for the paper and pencil version, the computerized LB values were also independently predicted by “odd beliefs or magical thinking,” and no significant correlation was found with MNL as a DV. The MNL result contradicts previous findings by Brugger and colleagues, who found in a sample of HS that leftward bias in number space is modulated by magical ideation: higher Magical Ideation scores were associated to a stronger leftward bias. According to the authors, this correlation may be explained in terms of an overreliance on a right hemisphere semantic system, which may lead to the association between magical thinking and lateral spatial attention (Brugger et al., 2010). Conversely, in a recent study we showed that SCZ expressed the same leftward bias in the visuo-spatial representation of numbers as HS (Ribolsi et al., 2013), confirming the previous findings of Tian and colleagues of a dissociation in performance between visual line and number bisection in schizophrenia (Tian et al., 2011).

Such dissociation between the two types of visuo-spatial bisection tasks (perceptual *vs*. representative) in SCZ may imply that the neural mechanisms underlying these different forms of pseudoneglect are not identical. Indeed, neuroimaging studies revealed that the visual LB task is related to the activity of the striate and the extrastriate visual cortex and of the parietal lobe (Husain and Nachev, 2007); in contrast, the mental number bisection task is mostly related to the prefrontal cortex beside the right parietal lobe (Rusconi et al., 2011; Tian et al., 2011). Furthermore, other authors have reported that representational forms of neglect only occasionally coexist with neglect in physical space (Loetscher et al., 2010). Moreover, neuropsychological examination revealed that the apparent left-sided neglect in the bisection of number intervals has a purely non-spatial origin (Van Dijck et al., 2011). Interestingly, it has recently been shown that bias toward higher numbers in the mental bisection of number intervals in right brain-damaged patients depends on disruption of a purely abstract non-spatial representation of small numerical magnitude (Aiello et al., 2013), confirming the hypothesis that perceptual and representational pseudoneglect have different underlying neurobiological substrates.

Therefore, in our study we hypothesize that, unlike the LB test, performance in the MNL test is not influenced by schizotypy, probably because of its non-spatial origin and because it involves different neural circuits from those of perceptual pseudoneglect (Van Dijck et al., 2011). This conclusion may be in line with the suggestion that schizophrenia spectrum disorders should be seen as the consequence of basic perceptual anomalies (Silverstein et al., 2000; Herzog et al., 2004; Uhlhaas and Mishara, 2007; Silverstein and Keane, 2011) rather than as “representational disorders.”

## Conclusion

The main finding of this study is that a decreased pseudoneglect as assessed by the LB task correlates with positive schizotypy in the healthy population. This result is in line with our previous study (Ribolsi et al., 2013), where we found a lack of normal leftward bias in a sample of SCZ. Taking the two studies together, we can hypothesize the existence of a correlation between the deviation from the leftward bias in the LB task and the degree of psychotic traits across the population. Deeper analysis is required, however, of a number of areas. First, LB performance should be investigated in a sample of patients with a diagnosis of schizotypal personality disorder. One possibility is that such patients may display intermediate behavior between HS with schizotypal traits and schizophrenia patients, but this hypothesis should be demonstrated in a specific study. Second, further research may be needed to relate measures of pseudoneglect with factors of schizotypy rather than the single dimensions of SPQ, as we did in this paper. Third, the neurobiological underpinning of the correlation between the degree of pseudoneglect and the severity of schizotypal traits in the healthy population should be studied. In the case of schizophrenia patients, more substantial data may suggest a pivotal role of the right parietal cortex dysfunction (McCourt et al., 2008; Ribolsi et al., 2013) to explain the lack of normal leftward bias in the LB test, but in a healthy population with schizotypal traits it is more difficult to draw any definitive conclusions.

### Conflict of interest statement

The authors declare that the research was conducted in the absence of any commercial or financial relationships that could be construed as a potential conflict of interest.
