# Supplementary material for: Negative correlation between leftward bias in line bisection and schizotypal features in healthy subjects
Source: Front Psychol. 2013 Nov 14;4:846. doi: 10.3389/fpsyg.2013.00846 (PMC3827540; doi:10.3389/fpsyg.2013.00846)
Supplement: Supplementary file 1 [file DataSheet1.PDF]

SUPPLEMENTARY MATERIALS

Table 1 and Table 2 summarize descriptive statistics and univariate analysis (*t*-test for independent sample and Pearson's product-moment correlation) of Experiment 1 and Experiment 2.

Table 1. Descriptive statistics and p-value of *t*-test for independent sample in Experiment 1 and Experiment 2.

	Experiment 1				Experiment 2			
	Total sample (n = 205)	Women (n = 136)	Men (n = 69)	p	Total sample (n = 80)	Women (n = 46)	Men (n = 34)	p
Age	34.78 (12.74)	35.04 (13.41)	34.44 (12.35)	0.798	32.23 (11.05)	32.51 (11.41)	31.93 (10.26)	0.780
EHI	59.14 (35.04)	63.80 (30.25)	51.52 (41.09)	0.113	57.25 (35.43)	63.80 (30.25)	51.52 (41.09)	0.113
Ideas of references	1.54 (1.88)	1.70 (1.94)	1.28 (1.77)	0.191	1.69 (1.97)	1.86 (2.00)	1.40 (1.89)	0.220
Excessive social anxiety	2.03 (1.91)	2.32 (1.89)	1.56 (1.87)	0.021	2.01 (1.90)	2.39 (1.93)	1.36 (1.68)	0.003
Odd beliefs or magical thinking	0.66 (1.13)	0.77 (1.15)	0.48 (1.08)	0.136	0.68 (1.13)	0.75 (1.12)	0.56 (1.16)	0.364
Unusual perceptual experiences	1.04 (1.34)	1.18 (1.39)	0.80 (1.22)	0.096	1.10 (1.37)	1.22 (1.40)	0.89 (1.30)	0.195
Odd or eccentric behaviour	1.08 (1.74)	0.86 (1.61)	1.44 (1.89)	0.063	1.16 (1.79)	0.97 (1.70)	1.47 (1.91)	0.144
No close friends	0.88 (1.49)	0.73 (1.42)	1.13 (1.58)	0.119	0.88 (1.45)	0.76 (1.37)	1.07 (1.57)	0.268
Odd speech	2.15 (2.28)	2.07 (2.12)	2.28 (2.53)	0.596	2.11 (2.19)	2.04 (2.04)	2.22 (2.45)	0.660
Constricted affect	1.08 (1.47)	1.00 (1.40)	1.22 (1.59)	0.384	1.06 (1.39)	1.01 (1.35)	1.13 1.46)	0.647
Suspiciousness	1.39 (1.52)	1.48 (1.72)	1.24 (1.12)	0.368	1.49 (1.58)	1.61 (1.79)	1.29 (1.14)	0.290
LB index	-0.10 (0.27)	-0.13 (0.28)	-0.06 (0.26)	0.135	-0.09 (0.28)	-0.12 (0.29)	-0.04 (0.27)	0.165
MNL index	–	–	–	–	-0.09 (0.29)	-0.11 (0.30)	-0.06 (0.27)	0.422

Table 2. Descriptive statistics and Pearson's product-moment correlation coefficient (r) in Experiment 1 and Experiment 2.

	Experiment 1	Experiment 2	
	LB index	LB index	MNL index
Age	0.140*	0.103	0.085
EHI	-0.209**	-0.221**	-0.119
Ideas of references	0.050	0.061	0.047
Excessive social anxiety	0.003	-0.001	-0.044
Odd beliefs or magical thinking	0.217**	0.234**	0.123
Unusual perceptual experiences	0.030	0.027	0.039
Odd or eccentric behaviour	0.054	0.073	0.049
No close friends	0.099	0.115	0.034
Odd speech	0.133	0.101	0.081
Constricted affect	-0.027	-0.060	-0.071
Suspiciousness	-0.001	-0.014	-0.007

* $p < 0.05$
** $p < 0.01$
